# Vorhofflimmerndiagnostik mittels EKG-fähiger Smartwatches

**DOI:** 10.1007/s00115-021-01124-x

**Published:** 2021-04-30

**Authors:** Wilhelm Haverkamp, Olaf Göing, Markus Anker, Stefan D. Anker

**Affiliations:** 1 Abteilung für Kardiologie und Metabolismus, Med. Klinik für Kardiologie, Campus Virchow-Klinikum, Charité – Universitätsmedizin Berlin, Augustenburger Platz 1, 13353 Berlin, Deutschland; 2 Sana Klinikum Lichtenberg, Berlin, Deutschland; 3 Berlin Institute of Health Center for Regenerative Therapies (BCRT), Berlin, Deutschland

## EKG-fähige Smartwatches

Die Möglichkeit, jeder Zeit und beliebig oft ein 1‑Kanal-Elektrokardiogramm (EKG) registrieren zu können, ist eine relativ neue Funktionalität von Smartwatches, die anhaltend für Aufmerksamkeit sorgt. Aus ärztlicher Sicht interessiert vor allem die Frage, ob es Sinn macht, EKG-fähige Smartwatches routinemäßig im Rahmen der Diagnostik von asymptomatischem Vorhofflimmern einzusetzen. Ein solches Screenings zielt darauf ab, identifizierte Patienten frühzeitig (ein erhöhtes Schlaganfallrisiko vorausgesetzt) einer dauerhaften oralen Antikoagulation zuzuführen [1]. In diesem Zusammenhang ist wichtig, dass ein mittels Smartwatch-EKG dokumentiertes, 30 s anhaltendes Vorhofflimmern (die Registrierdauer beträgt max. 30 s) laut aktueller Leitlinien dann diagnostisch verwertbar ist, wenn die Diagnose von einem Arzt bestätigt wurde – eine erneute Bestätigung der Diagnose durch ein weiteres EKG-Verfahren (z. B. ein Langzeit-EKG) ist nicht notwendig [1].

## Algorithmen und Arbeitsweise

EKG-fähige Smartwatches sind mit Software-Applikationen (Apps) ausgestattet, die es erlauben, Vorhofflimmern automatisch zu diagnostizieren. Andere Rhythmusstörungen werden nicht automatisch erkannt. Ein Teil der Smartwatches ermöglicht darüber hinaus ein Vorhofflimmernscreening mittels photoplethysmographischer Pulswellenanalyse. Wenn sich hierbei der Verdacht auf Vorhofflimmern ergibt, wird der Patient via App benachrichtigt und gebeten, ein EKG zu registrieren. Die bislang zur Validierung dieser neuen Techniken zur Verfügung stehenden Daten reichen für eine endgültige Bewertung ihrer diagnostischen Wertigkeit unter Real-world-Bedingungen nicht aus [1]. Im Vorfeld zu erwartender Studienergebnisse erschien es den Autoren dieses Beitrages sinnvoll, die Algorithmen und Arbeitsweisen EKG-fähiger Smartwatches zu analysieren (Tab. 1). Hierzu wurden die im Rahmen von Zulassungsverfahren und der Bewerbung öffentlich gemachten Informationen zur EKG-Funktion ausgewertet [2–6]. An diese Informationen heranzukommen, erwies sich zum Teil als sehr aufwendig. Die Politik ist gefordert, Standards zu schaffen, die es Ärzten erlaubt, neue Techniken (die als Medizinprodukte zugelassen sind) unter medizinischen Gesichtspunkten realistisch bewerten zu können.

**Table 1:** 

Hersteller/Produkt	Automatische VHF-Erkennung	VHF-Screening	Kommentare
*Apple* Apple Watch Series 4, 5 und 6	Version 2.0: 50–150/min	+	EKG-App und VHF-Screening-App von der FDA freigegeben (08/2018) und CE-zertifiziert (03/2019). Aktualisierte EKG-App (Vers. 2.0) seit 01/2021 verfügbar
	Version 1.0: 50–120/min		
*Fitbit* Sense	50–120/min	–	Von der FDA freigegeben (08/2020) und CE-zertifiziert (08/2020). Seit 10/2020 in D im Handel. VHF-Screening wird gerade geprüft
*Samsung* Galaxy Watch Active 2, Galaxy Watch 3	50–120/min	(+)^a^	Von der FDA freigegeben (09/2020) und CE-zertifiziert. Wohl ab 03/2020 auch in Deutschland verfügbar
*Withings* Move ECG, Scanwatch	50–120/min^b^	+	Move ECG: CE-zertifiziert (06/2019), Scanwatch: CE-zertifiziert (06/2020). FDA-Freigabe ausstehend

*VHF* Vorhofflimmern, *FDA* Food and Drug Administration, *CE* Conformité Européenne (Europäische Konformität)

^a^Nur Herzfrequenzalarm

^b^Angabe laut Bewerbung, laut Hersteller keine eigentliche Grenze, Daten zur Validierung stehen aus

## Diagnostische Genauigkeit

**Figure 1:**
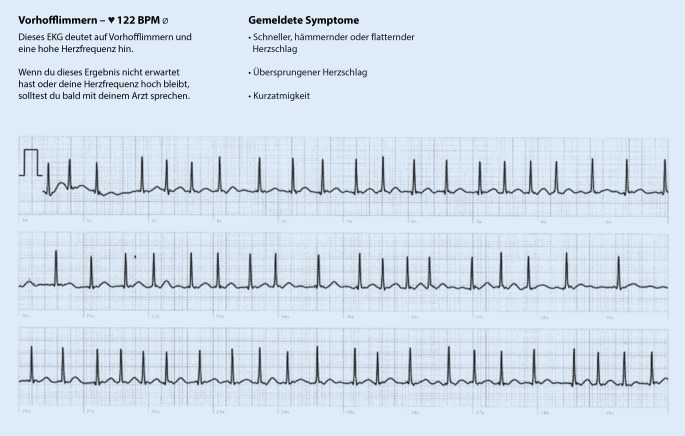

Wenn es um die reine Erkennung von Vorhofflimmern geht, liegen die für EKG-fähige Smartwatches mitgeteilten Sensitivitäten und Spezifitäten bei über 95 % [2–7]. Anzumerken ist, dass die Untersuchungen unter sehr standardisierten Bedingungen stattfanden. Darüber hinaus beziehen sich diese Angaben auf auswertbare EKGs – etwa 10–20 % der mit einer Smartwatch registrierten EKGs sind nicht auswertbar (z. B. bedingt durch Artefakte). Die in Tab. 1 zusammengestellten Daten deuten drauf hin, dass die diagnostische Genauigkeit im Alltag niedriger sein dürfte. Vorhofflimmern mit einer Kammerfrequenz unterhalb von 50/min wird von keiner der Smartwatches erkannt. Auch bei hohen Herzfrequenzen ergibt sich ein Grenzwert, ab dem nicht mehr auf Vorhofflimmern geprüft wird (Abb. 1 und 2). Dieser liegt bei den meisten Uhren bei 120/min. Eine überarbeitete Version des Apple-Algorithmus detektiert jetzt auch Vorhofflimmern mit einer Kammerfrequenz von bis zu 150/min [7]. Die Genauigkeit des Algorithmus ist jedoch bei Kammerfrequenzen von über 100/min deutlich niedriger als bei einer Kammerfrequenz von 50–99/min (98,3 % gegenüber 83 %; [7]). In diesem Zusammenhang ist es wichtig festzustellen, dass neu auftretendes Vorhofflimmern unter klinischen Bedingungen in etwa einem Drittel der Fälle Kammerfrequenzen von über 120/min aufweist, nicht selten liegt die Frequenz über 150/min [8].

**Figure 2:**
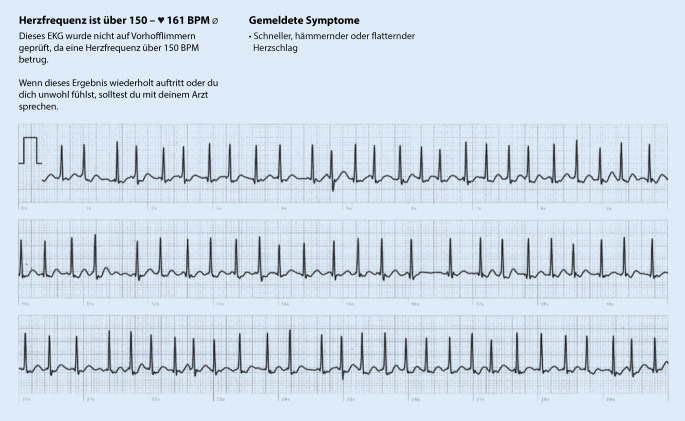

Beim Smartwatch-basierten Screening auf Vorhofflimmern muss berücksichtigt werden, dass die hierbei genutzten photoplethysmographischen Messungen – da sie viel Strom verbrauchen – nur in Ruhe und nur intermittierend erfolgen. Das Vorgehen unterscheidet sich herstellerabhängig. Es entstehen diagnostische Lücken, die dazu führen, dass insbesondere kurze Arrhythmieepisoden nur unzureichend erkannt werden. Für solche diagnostische Lücken spricht auch die relativ geringe Anzahl an Benachrichtigungen über Vorhofflimmern in der Apple-Heart-Studie, in die mehr als 400.000 Personen eingeschlossen wurden [9]. Eine Benachrichtigung über Pulsunregelmäßigkeiten via App erfolgte nur bei 0,52 % der Probanden. Mit 3,2 % war die Benachrichtigungsrate auch in der kleinen Subgruppe der über 65-Jährigen (*n* = 24,6326) relativ niedrig. Die Studie hat gezeigt, dass ein Smartwatch-basiertes Screening auf Vorhofflimmern prinzipiell möglich ist, die diagnostische Genauigkeit des Verfahrens lässt sich aber, da eine Positivkontrolle fehlte, nicht berechnen. Studien an kleinen Kollektiven, die eine relativ hohe Sensitivität zeigen, reichen für eine abschließende Bewertung nicht aus [10].

## Konsequenzen für den Alltag

Basierend auf den derzeit implementierten, je nach Hersteller unterschiedlichen Algorithmen bzw. Arbeitsweisen von Smartwatches bestehen, wie auch bei allen anderen EKG-Verfahren, Lücken bei der Diagnostik von Vorhofflimmern. Diese sollten dem Anwender und Auswerter von Smartwatch-EKGs bekannt sein. Eine Konsequenz ist z. B., dass bei EKG-Dokumentationen mit hohen Frequenzen (über 120 bzw. 150/min), die aufgrund der internen Algorithmen nicht automatisch auf Vorhofflimmern geprüft werden, vermehrt mit falsch-negativen Befunden gerechnet werden muss. Eine zukünftige Optimierung der Algorithmen durch den Einsatz von künstlicher Intelligenz ist denkbar [11, 12]. Eine genaue Kenntnis der Smartwatch-spezifischen Algorithmen wird besonders dann wichtig, wenn ärztlicherseits, basierend auf den Befunden, Entscheidungen getroffen werden.

## Fazit

- Smartwatch-EKGs stellen eine willkommene Erweiterung des Repertoires an diagnostischen EKG-Verfahren dar.
- Mittels Smartwatch dokumentiertes Vorhofflimmern ist diagnostisch verwertbar, sofern die Diagnose von einem Arzt bestätigt wurde.
- Aufgrund diagnostischer Lücken ist ein Ausschluss von Vorhofflimmern, wie auch bei anderen EKG-Verfahren, nicht möglich.
- Beim Einsatz EKG-fähiger Smartwatches in klinischen Studien muss klar sein, dass die Häufigkeit von Vorhofflimmern unterschätzt wird.
- Es ist anzunehmen, dass sich zukünftig, z. B. basierend auf künstlicher Intelligenz, eine verbesserte diagnostische Genauigkeit ergeben wird.
